# Factors associated with human papillomavirus infections among women living with HIV in public health facilities in Western Oromia, Ethiopia

**DOI:** 10.1186/s12905-024-03249-y

**Published:** 2024-07-25

**Authors:** Mulatu Abdi, Afework Tamiru, Temesgen Tilahun, Gemechu Tiruneh, Meseret Belete Fite

**Affiliations:** 1 Nekemte public health research and referral laboratory, Oromia regional health bureau, Nekemte, Ethiopia; 2 Department of Public Health, Institute of Health Sciences, Wollega University, Nekemte, Ethiopia; 3 School of medicine, Institute of Health Sciences, Wollega University, Nekemte, Ethiopia; 4 Department of Public, Sunshine College, Nekemte, Ethiopia

**Keywords:** Human papillomavirus, Women, Human immunodeficiency virus

## Abstract

**Background:**

Human Papillomavirus infection (HPV) is among the most common sexually transmitted infections with the highest incidence and prevalence worldwide. HPV has been established as the main cause of cervical cancer and remains a public health problem globally. In Western Oromia, Ethiopia cervical screening remains a major issue because of limited resources, and shortage of HPV testing technology. As a result, the prevalence of HPV and associated factors remain unknown among HIV-positive women. This study aimed to assess the prevalence of HPV and associated factors among women living with HIV attending Antiretroviral Therapy (ART) services in public health facilities of East Wollega and West Showa Zones, Ethiopia, 2022.

**Method:**

Using a cross-sectional study design, a total of 415 women ≥ 18 years old were enrolled using systematic random sampling from five public health facilities. Cervical specimens were collected by a trained nurse from April 01 2022, to May 30^,^ 2022, and tested at Nekemte Public Health Research and Referral Molecular Biology, a certified/accredited laboratory for HPV-DNA Polymerase Chain Reaction by expertise using Abbott m2000rt-PCR assays. Finally, Epi data version 4.6 was used for data entry and SPSS version 24.0 were used for data cleaning and analysis, and frequencies and prevalence of HPV were computed. Variables were identified using the multivariable model and statistically significant associations of variables were determined based on the adjusted odds ratio (AOR) with its 95% CI and P-value < 0.05 to determine the strength of association.

**Result:**

The prevalence of HPV was 30.4% [95% CI: 26.0, 34.9]. Of HPV-infected women, 11.9% were positive for HPV-16, 9.5% for HPV-18, and 65.9% were positive for other hr-HPV . The odds of HPV infection among women aged beyond 48 years are **2.85 times** the odds of HPV among people who were aged 18–27(AOR = 2.85, 95% CI: 1.16, 5.58). The odds of HPV infection among women who had three or more sexual partners is 4.12 times the odds of HPV infection among women with a single sexual partner(AOR = 4.12, 95% CI: 2.34–8.62). The odds of HPV infection among women who didn’t use condom during sexual intercourse are 4.73 **times** the odds of HPV among women who used condom during sexual intercourse. (AOR = 4.73, 95% CI: 1.98–9.33). The odds of HPV infection among women who had history of is 4.52 **times** the odds of HPV infection among women with no history of abortion. [AOR = 4.52, 95% CI: 2.04, 6.89] The odds of HPV infection among women with history of Sexually Transmitted Infection (STI) **3.62 times** the odds of HPV among women with no history of STI (AOR = 3.62, 95%CI: 1.75, 5.83). The odd of HPV among women with abnormal vaginal discharge is 3.31 times the odds of the disease among women with normal vaginal discharge [AOR = 3.31, 95% CI: 2.87,7.35).

**Conclusion and recommendation:**

The prevalence of HPV infection among HIV-infected women was high in the study area. Given the above-associated factors, we recommend that the stakeholders integrate HPV prevention strategies into HIV /AIDS services. Furthermore, the study has provided essential information about the HIV link with hr-HPV infections, which may explain the high prevalence among HIV-infected women. This can contribute to policy development and planning of prevention strategies incorporating HPV infection prevention especially among youth and HIV-infected people.

**Supplementary Information:**

The online version contains supplementary material available at 10.1186/s12905-024-03249-y.

## Background

Worldwide, human papillomavirus (HPV) is the most common sexually transmitted infection (STI) and a main reason for morbidity and mortality [1]. Several studies recommend that 75% of all sexually active people will become infected to a certain extent in their lifespan [2, 3]. At present, more than 120 HPV types have been recognized, and tenacious infection with hr-HPV genotypes is related to the advance of cervical intraepithelial lesions leading to cervical cancer [3]. Majority of the exposures do not result in infection and resolve instinctively. Grounded on the occurrence of detection in CC, HPV genotypes are categorized into high-risk HPV (Hr HPV) types (16, 18, 31, 33, 35, 39, 45, 51, 52, 56, 58, 59, 66 and 68) and low-risk (LR) types (6, 11, 42, 43, 44, 54, 61, 70, 72, and 81**)** [4]. Hr HPV triggers nearly all cases of cervical cancer. HPV types 16 and 18 are the most common types of Hr HPV and contribute to about 70% of cervical cancer which commonly originates in the cells of cervix [5–7].

Cervical cancer is the fourth most frequently diagnosed cancer and the fourth leading cause of cancer death in women, with an estimated 604,000 new cases and 342,000 deaths worldwide in 2020 [8] It is still the most common female cancer globally and HPV has an incidence rate of 24.1 per 100, 000 women and years in Eastern African countries leaving it the leading cause of cancer deaths among females with an estimated overall prevalence of two-fold higher (29%) with normal cervical cytology [9, 10]. Cervical cancer as a result of HPV infection in Ethiopia was also high among women of ≥ 15 years, and 31.5 million are at risk with annual cases of 6,294 and deaths of 4,884 and an incidence rate of 11.7 per 100,000 women and year [11].

The burden HPV prevalence has been found to differ across the countries, for instance, 52.9% in South Africa, 55.5% in Botswana, Democratic Republic of Congo 31.3%, and 43.6% in Zimbabwe: these differences could be due to many factors, including different HPV detection assays, the type of specimen used, the age of the study population, presence of HIV co-infection, and characteristics of the study population. In Ethiopia, a systematic review and meta-analysis conducted in 2019 (from cross-sectional VIA and Cytology method) on the prevalence of cervical lesions among Ethiopian regions show 15.1% in Addis Ababa, 19.65% in Southern nation and nationalities region, 14.35% in Amhara and 12.9% in others and 15.27% particularly among HIV positive [10, 12–16]. Due to HPV infection cervical cancer became the 2nd most common leading cause of cancer deaths next to breast cancer in women aged 15 to 44 years in Ethiopia with a significant incidence rate of 11.7 during the period per 100,000 women and year [11, 12]. The HPV vaccine was introduced in Ethiopia in 2018, targeting 14-year-old girls. However, recent study highlighted low level of knowledge about the HPV vaccine and sub-optimal uptake of vaccination which falls short of the 2030 WHO targets [17].

Despite the existing evidences suggesting the high burden of HPV among women living with HIV, cervical cancer screening coverage is still limited. This problem is more pronounced in the current study area due to existing conflicts which has dramatically impacted the health care service delivery. Study on HPV prevalence is also fragmented and factors associated with infection are not well investigated [11]. The aim of this study was to assess the prevalence of the Human papillomavirus and identify associated factors among women living with HIV attending Anti- Retroviral Therapy (ART) services in public health facilities of East Wollega and West Showa Zones, Ethiopia during the study period using HPV-Deoxy Ribonucleic acid *real-time* Polymerase Chain Reaction technology.

## Methods

### Study settings and period

The study was conducted among women living with HIV attending ART services in public health facilities of Western Oromia, Western Ethiopia. Western Oromia has Six [5] zones having an estimated total population of 12,357,510. Among six zones, two zones; West Showa and East Wollega Zonal administration were randomly selected. There were five ART clinics Public Hospitals and seven ART clinics Health center facilities with trained health care providers and necessary supplies and equipment providing cervical cancer screening services. As a new initiation to respond to the World Health Organization (WHO) call for all countries to work towards the elimination of cervical cancer as a public health problem the Ministry of Health (MOH) Ethiopia started cervical cancer screening service for women living with HIV who are vulnerable to Human Papilloma virus and at greater risk of developing cervical cancer. Ambo General Hospital, Nekemte Specialized Hospital, Enchini Health Center, Ginch Health Center, and Nekemte Health Center were selected. This study was conducted from January 5 to February 12, 2022.

### Study design

An institutional-based cross-sectional study design was deployed to achieve the objective of the study.

### Source population

All women living with HIV attending ART services in public health facilities of Western Oromia were the source population for this study. Women living with HIV attending ART services in the selected public health facilities who were included in the study were the study population.

### Sample size determination and sampling procedures

**Fig. 1:**
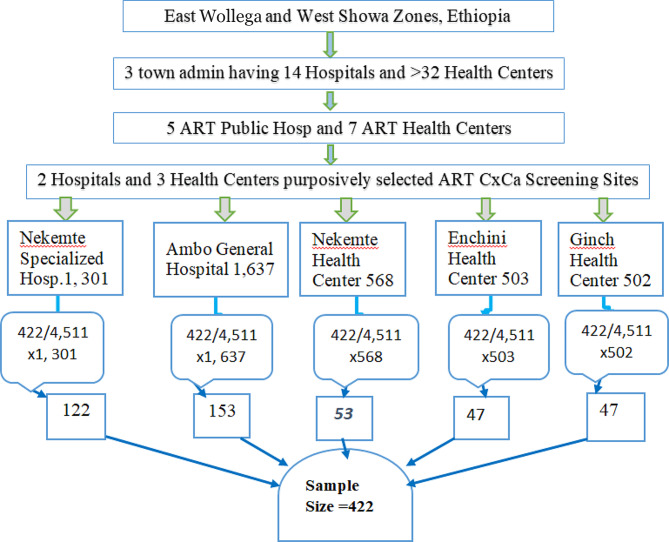
Schematic representation of sampling technique

The required sample size was calculated by the single population proportion formula by considering proportion [P] to be 50% assuming the lack of a study with a similar detection method in Ethiopia. A sample size of 384 subjects was primarily determined using the formula and, further increased by adding a 10% non-response rate making the final sample size to be 422 HIV-positive women aged 18 years and above. The sample size was proportionally recruited across the health institutes based on the size of the source population for of each the selected facilities. After preparations of a sampling frame, the study subjects were selected using systematic random sampling technique until attainment of the predetermined final sample size (Fig.1).

### Data collection tools and techniques

Two sets of tools were used to collect data in this study. Structured questionnaire was administered to the study participants for collection of a primary data addressing the socio-demographic, socio-economic and behavioral attributes of the study subjects. The secondary data such as clinical correlates were extracted using a data extraction checklist purposed for the study from a clinical registry of the patients. The structured questionnaire was prepared in English (Supplementary file 1), translated to Afaan Oromo, and translated back to English to verify its consistency.

### Biochemical measurement

The cervical sample bio specimen was collected from the study subjects by trained health professionals who provided the screening program. The collected samples are precisely labeled with a necessary information, placed in sample tubes containing a liquid transportation medium for preserving the sample and stored at 2-8^O^ C until shipment to the central laboratory. All cervical samples collected were registered on a sample tracking sheet and transported to the testing center; Nekemte Public Health Research and Referral Laboratory [NPHRRL] in a triple package maintained cold chain. The laboratory is a nationally accredited by Ethiopian National Accreditation Office [ENAO] for extraction of DNA genomes. The cervical samples were tested at a molecular biology laboratory certified/accredited, Nekemte Public Health Research and Referral Laboratory].

DNA genomes from the cervical samples were extracted by a fully automated robotic Abbott m2000sp machine [Abbott, USA]. The quality control for the procedure was monitored by commercially prepared negative and positive controls kits throughout the sample processing, the amplification, and the detection steps. The test was performed as per the manufacturers guide for HPV-DNA in a real-time PCR assay using Abbott m2000rt PCR machine.

Three HPV signals corresponding to HPV-16, HPV-18, and other high-risk-HPV were evaluated for each sample. Each signal was either determined as “Detected” or “Not detected” for all the three category signals. Samples with any of the 3 high-risk-HPV signals detected have an interpretation of “high risk-HPV Detected which is reported as a positive result and, signals with no HPV genome detected were reported as a ‘’negative”. All procedures from sample collection, storage, processing and, laboratory analysis were performed as per the standard of WHO [7].If any of the three high-risk-HPV signals are recovered in the specimens, it is reported as detected which is interpreted as “high risk-HPV Detected” and this is equated as a negative test result. If the high risks HPV are not recovered from the specimen it is reported as not detected and ultimately reported as a negative test result. All subjects’ results were confidentially communicated to the respective health facilities for an intervention.

### Data quality assurance

The quality of data was assured by properly designing and pre-testing the questionnaire and properly training the data collectors and supervisors on the data collection procedures. Two days of rigorous and extensive training with the final version of the questionnaires were given to each data collector and supervisor before the pre-test. Supervisors checked collected data before being sent to the data entrée on a daily basis. Questionnaires were pre-tested on 10% of the sampled women living with HIV attending ART service in the Gimbi Hospital, that were not included in the main study, and modification was done based on the pre-test observations. The necessary feedback is offered to data collectors before the actual procedure. The supervisors kept the alleyway of the field procedures. They checked the completed questionnaires daily to approve the accuracy of the data collected, and the research team managed the overall work of data collection. Proper storage temperature, sample tracking to the testing laboratory, and standard operating procedures in molecular testing as per quality policy were all maintained.

### Data processing and analysis

Data was entered using Epi-data 3.1. The filled data was exported to Stata 14 [College Station, Texas 77,845 USA] for analysis. Data were checked for missing and outlier value, cleaned, coded, and analyzed by Stata 14. Software. All variables were described as frequency and percentages. Data was presented as tabular and in charts. The outcome variable; the prevalence of Human Papilloma Virus was dichotomized as Human Papilloma Virus positive or Human Papilloma Virus negative. Then binary logistic regression was employed for each independent variable against the outcome variable to identify candidate variables for multivarable analysis. All variables with a *p* < 0.25 in the binary logistic regression were transferred to multiple logistic regressions. Adjusted odds ratios [AOR] at 95% CIs was used to identify the significantly associated-factors with the estimated prevalence of HPV. Multi-collinearity was checked by computing the variance inflation factor [VIF] value to check existence of correlation between independent variables. No evidence of correlation existed among the variables as per the estimated VIF value which was below 10. The model’s goodness-of- fit was assessed using Hosmer-Lemeshows. Backward elimination was used and p-<0.05 was considered statistically significant level of declaration.

### Ethical considerations

This study was conducted in agreement with the Declaration of Helsinki-Ethical principle for medical research involving human subjects. Before starting of the data collection process, the proposal was approved by the Wallaga University Research Ethics Review Committee [ref: WUIHS/022/2022]. Letter of cooperation was written to the facilities participating in the study and their respective focal persons before commencement of the data collection. Written informed consent was obtained from all study participants and confidentiality was maintained by excluding all personal identifiers.

## Results

### Socio-demographic characteristics of the study participants

A total of 415 women living with HIV were actually enrolled to the study. This makes up a response rate of 98.3% compared to priory determined sample size. The mean age of the women was 35.36 years with a standard deviation of 6.77. Half of the study participants appeared to exist within the age group of 28–37. Regarding their marital and religious status, just over half of them were married [53%] and protestant [55%] respectively. The vast majority of the respondents were identified as belonging to the Oromo ethnic group [85.5%] and a similar amount [82.4%] reported as urban residents (Table 1).

**Table 1:** Socio-demographic characteristics of women living with HIV attending ART service in public health facilities of Western Oromia, Western Ethiopia, 2022 [*n* = 415]

Variables	Category	Frequency[*n* 415]	Percentage [%]
Age [years] Mean [ *±* SD]		35.36[*±* 6.77]	
Age Group	18–27	55	13.3
	28–37	209	50.4
	38–47	130	31.3
	>48	21	5.00
Residence	Urban	342	82.4
	Rural	73	17.6
Ethnicity	Oromo	355	85.54
	Amhara	45	10.84
	Gurage and Tigre	15	3.61
Religion	Orthodox	161	38.8
	Protestant	228	54.9
	Muslim	19	4.6
	Catholic	7	1.7
Marital status	Single	45	10.8
	Married	220	53.0
	Divorced	96	23.1
	Widowed	54	13.0
**Educational level**	No formal education	128	30.8
	Primary [1–8]	174	41.9
	Secondary[ 9–10]	56	13.5
	High school [11–12]	20	4.8
	Tertiary level	37	8.9

### Prevalence of HPV infection and, genotype distribution

**Fig. 2:**
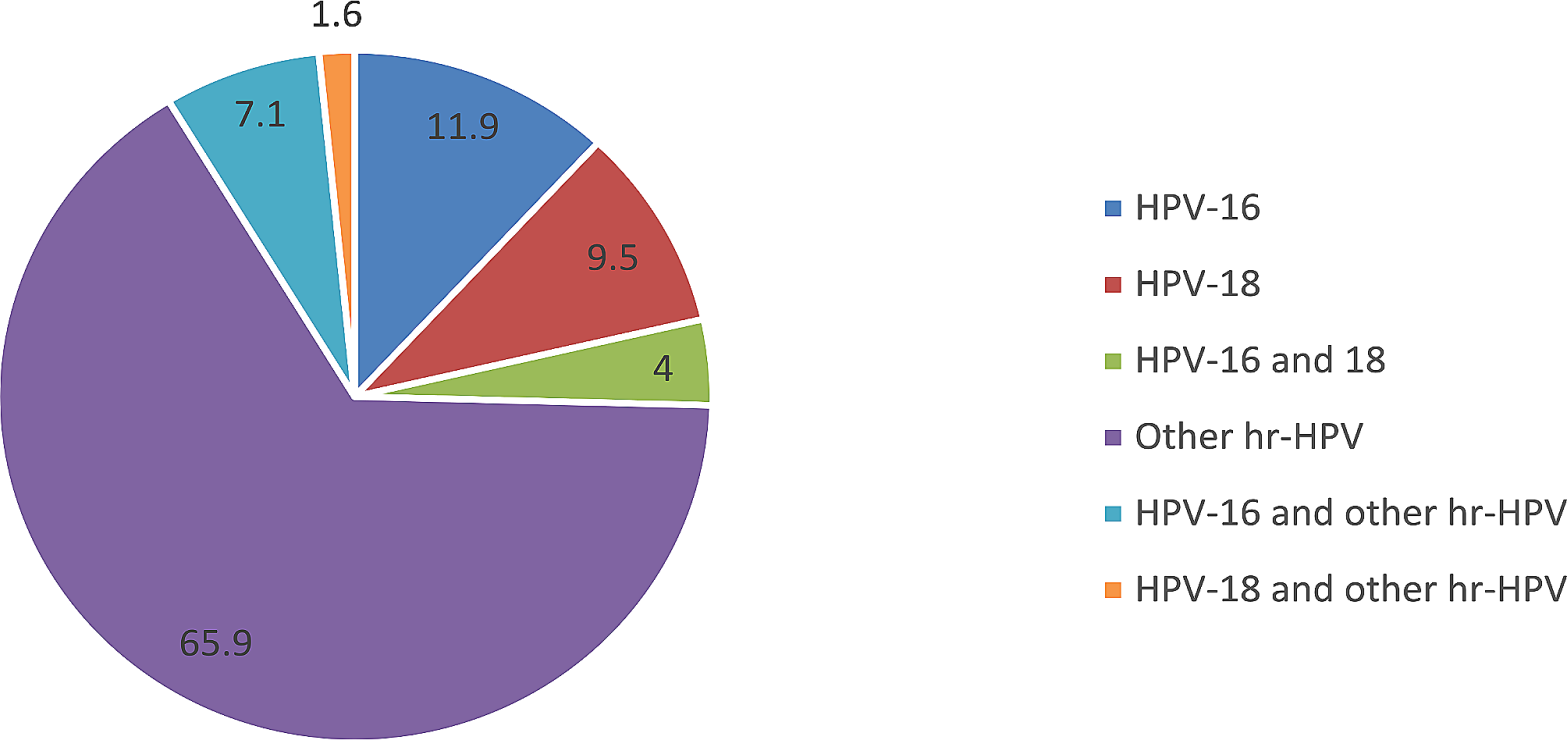
The prevalence of HPV infection and its genotype distribution among women living with HIV attending ART service in public health facilities of Western Oromia, Western Ethiopia, 2022 (*n* = 415)

The overall prevalence of HPV was 30.4% [95%CI: 26.0, 34.9]. Regarding the genotype distribution HPV infection 11.9% were HPV-16 positive, 9.5% positive for HPV-18, 65.9% positive for other Hr-HPV types, 4.0% positive for HPV-16 and 18, 7.1% positive for HPV-16 and other Hr-HPV and 1.6% were positive for HPV-18 and other Hr-HPV types (Fig.2).

### Sexual history, fertility, and clinical correlates of the study participants

Age at marriage for the majority [82.4%] of the study participants were 18 years and beyond. For the majority of the participants, [82.3%] the age of first menstrual cycle experience was beyond 13 years of age. Beyond half [60%] of the study participants reported the first sexual debut being beyond age of 18. Similarly, almost equal amount [58.3%] of them conceived first pregnancy in their twenties. Study participants who had 0–2 pregnancy accounted for 62.9% of the whole. Participants who experienced miscarriages and who reported non-use of condoms during sexual intercourse were seemingly equal which is 34.2% and 33.3% respectively. Nearly half [47.2%] of the study participants had one sexual partner and the remaining of them had two or three sexual partners during their lifetime. Although the majority [65.1%] of the participants did not remember they had a diagnosis of STI in the last year, one-third of them confirmed a recent history of other STIs. Close to 90% [88.5%] of them were at WHO clinical staging of I or II sharing equal proportions (Table 2).

**Table 2:** Fertility and clinical characteristics of women living with HIV attending ART service in public health facilities of Western Oromia, Western Ethiopia, 2022 [*n* = 415]

Variables	Category Variables	Frequency[*n*]	Percentage [%]
Age at Menarche [years]	10–12	73	17.6
	> 13	342	82.4
Age at Marriage [years]	< 15	12	2.9
	15–17	56	13.5
	> 18	347	83.6
Age at first sexual intercourse [years]	< 16	56	[13.5
	16–18	109	26.3
	> 18	250	260.2
Age at first pregnancy [years]	20–29	242	58.3
	> 30	50	12.0
	< 19	123	29.6
Number of pregnancy	0–2	261	62.9
	3–5	141	34.0
	> 6	13	3.1
Abortion/Miscarriage	Yes	142	34.2
	No	273	65.8
Condom use during sexual intercourse	Yes	138	33.3
	No	277	66.7
Number of Life time partners	1	196	47.2
	2	135	32.5
	3+	84	20.2
Contracted STIs in the last 12 months	Yes	122	29.4
	No	23	5.5
	Don’t know	270	65.1
WHO HIV Clinical stage	Stage 1	187	45.1
	Stage 2	180	43.4
	Stage 3	48	11.6

### Factors associated with HPV infection among the study participants

A two-phase approach of model building was considered to identify the factors associated with the outcome variable. The bivariable model was a starting step of the model building aimed to identify potential candidate variables to the final model. In the bi-variable analysis, the age of respondents, marital status, educational level, use of condom, number of sexual partners, gravidity, history of abortion, History of STIs, Abnormal vaginal discharge, and clinical stage of HIV were found to be candidates for multivariable analysis at a p-value < 0.25. In the multivariable logistic regression model, age, number of lifetime sexual partners, use of condom during sexual intercourse, history of abortion, history of STIs, abnormal vaginal discharge and clinical stage of HIV showed a statistically significant association with risk of HPV among women living with HIV attending ART service.

The odds of HPV infection among women aged beyond 48 years are **2.85 times** the odds of HPV among people who were aged 18–27(AOR = 2.85, 95% CI: 1.16, 5.58). The odds of HPV infection among women who had three or more sexual partners is 4.12 times the odds of HPV infection among women with a single sexual partner(AOR = 4.12, 95% CI: 2.34–8.62). The odds of HPV infection among women who didn’t use condom during sexual intercourse are 4.73 **times** the odds of HPV among women who used condom during sexual intercourse. (AOR = 4.73, 95% CI: 1.98–9.33). The odds of HPV infection among women who had history of is 4.52 **times** the odds of HPV infection among women with no history of abortion. [AOR = 4.52, 95% CI: 2.04, 6.89] The odds of HPV infection among women with history of Sexually Transmitted Infection (STI) **3.62 times** the odds of HPV among women with no history of STI (AOR = 3.62, 95%CI: 1.75, 5.83). The odd of HPV among women with abnormal vaginal discharge is 3.31 times the odds of the disease among women with normal vaginal discharge [AOR = 3.31, 95% CI: 2.87,7.35) (Table 3).

**Table 3:** Factors associated with HPV infection among women living with HIV attending ART service in public health facilities of Western Oromia, Western Ethiopia, 2022 [*n* = 415]

Variables	Variable Category	HPV status [positive/whole]	COR [95%CI]	AOR[95%CI]	*P*-value
**Age**	18–27	10/55	1	1	
	28–37	62/209	1.89[0.89,4.01]	2.08[0.53,8.11]	0.29
	38–47	43/130	2.22[1.02,4.84]	1.86[0.47,7.34]	0.37
	*≥* 48	11/21	4.95[1.65,14.82]	2.85[1.16,5.58]	**0.033**
Condom Utilization	Yes	30/138	1	1	
	No	96/277	1.91[1.18,3.07]	4.74[1.98,11.33]	0.001
Number of sexual partners	1	1	1	1	
	2	37/135	2.22[0.99,4.98]	1.95[0.52,7.29]	0.32
	3+	34/84	3.02[1.29,7.03]	4.12[2.34,8.62]	0.002
Gravidity	0–2	73/261	1	1	
	3–5	46/141	1.25[0.80,1.94]	1.42[0.64,3.17]	0.38
	≥ 6	7/13	3.01[0.98,9.24]	1.08[0.19,6.04]	0.92
History of abortion	Yes	94/142	14.75[8.89,24.48]	4.52[2.04,6.89]	0.001
	No	32/273	1	1	
History of STI	Yes	81/122	12.32[7.41,20.48]	3.62[1.75,5.83]	0.001
	No	45/275	1	1	
Abnormal vaginal discharge	Yes	82/151	5.94[3.77,9.37]	3.31[2.87,7.35]	0.001
	No	44/264	1	1	
Clinical Stage of HIV	Stage I	35/187	1	1	
	Stage II	68/180	2.64[1.64,4.24]	4.78[2.06,10.07]	0.01
	Stage III	23/48	3.99[2.03,7.85]	4.37[1.32,8.54]	0.016

## Discussion

This study assessed the prevalence, genotype distribution, and related associated factors of HPV infection among women living with HIV in public health facilities of western Oromia, western Ethiopia. The overall prevalence of HPV infections was 30.4% [95% CI: 26.0, 34.9]. Being older age, having multiple sexual partners, having a history of abortion, failing to use condoms during sexual intercourse, and having a history of STIs other than HIV and increased clinical stage of HIV were positively associated with HPV infection.

Our study found that more than a quarter of women living with HIV were infected with HPV which is comparably lower than report of previous study conducted in Shashemene town Southern Ethiopia (40%) [18]. However, this finding is higher than the previous studies in Uganda and other African countries where the prevalence ranges between 17% and 25% [12, 19–21]. The high prevalence in this country is a negative reflection of HPV vaccination programs, which have been largely ineffective due to underprivileged set-up and meagre finance [22]. Advanced countries such as Australia, which have exceedingly effective HPV vaccination programs, have lessened HPV infection rates to as low as 2.3% [23]. For cervical cancer to be eliminated it is a essential to encourage vaccination programs to reach at least 90% of girls below 15 years of age, as suggested by the WHO [24]. This is serious, particularly in SSA countries such as Ethiopia, where most deaths are due to cervical cancer.

In the present study, the most common genotypes were the other high-risk HPV genotypes (31, 33, 35, 39, 51, 52, 56, 58, 59, 66, and 68), followed by HPV-16 positive and positive for HPV-18. These results are in line with studies that conducted similar molecular techniques of hr-HPV DNA detection, such as results documented in Uganda [25], where the majority (63.89%) of the participants had other high-risk HPV, 13.89% had HPV 18/45 2.78%, in Swaziland [26], where the majority (45.3%) of the participants had other high-risk HPV, 12.4% had HPV 16 and 13.8% had HPV 18/45, and in India [27], where the majority (33.3%) had other high-risk HPV and 16.67% had HPV 18/45. However greatest cervical cancer cases have been formerly recognized to HPV 16 and 18, there is today rising indication on the significance of other high-risk HPV serotypes in cervical cancer causation. As more data is produced, there will be a crucial appraisal of vaccination policies to avail the nonavalent vaccine in all locales.

In the current study, infection of HPV was predominant among women of older age, with a trend to increased odds with increasing age. This is inconsistent with studies described in Brazilian cities [28–31]. The present study also show that Women Living with HIV(WLWH )who had more than three lifetime sexual partner were more likely to be infected with HPV than those WLWH who had less than three-lifetime sexual partner. A history of multiple sex partners is an important risk factor for the acquisition of HPV because the higher the numbers of partners drive more probability to interact with a contaminated partner. Having multiple sexual partners in life was also associated with an increase in the risk of HPV. This finding is in agreement with a similar study conducted in the Ethiopia [32], in Uganda [25], rural Eastern Cape of South Africa [33], Denmark [13], and Brazil [34]. This could be because the higher the number of partners drives more probability to interact with a contaminated partner [35, 36]. The possible explanation for this is that there are more than 200 different genotypes of HPV, which spread from one person to another by sexual contact, increased risk of getting HPV when there is sexual contact with multiple sexual partners.

In the present finding, there were strong associations between HPV infection and the use of condoms. Study participants who did not use condom during sexual intercourse were risk for developing HPV. Many studies have shown similar data [25, 37, 38]. This could be due to the regular use of condoms does not seem to provide total protection against microbial infection, given that the condom does not fully cover the male reproductive organ, hence leaving areas potentially harboring infectious particles or subclinical lesions, in the case of HPV, exposed during sexual intercourse associated to this perception we can observe the possibility that not all patients given the true response about his real behavior inserting a trend in this analysis that we cannot measure but is prudent to consider.

Having a history of STI was identified as a predictor of HPV infections. In the current study, women living with HIV who had a history of STI were significantly associated with HPV infection and it is in line with studies carried out in Ethiopia [32], Uganda [25] and Brazil [39]. The possible reason of this might be classically due to HPV infection being one type of STI and having others, type of STI is vital for entry and persistence.

Our result on relatively high rates of HPV infection among women with abnormal vaginal discharge is in line with those found in Uganda [25]. Participants who had a history of abnormal vaginal discharge as a result of STIs and had repeated history of abortion/miscarriage were also at higher risk to develop HPV infection. The loss of protecting microorganisms and other changes brought on by vaginal infections could make it easier for other STIs, such as HPV, to be easily acquired and persist. Clinicians managing patients should consider performing HPV screening on them.

In our study, being HIV positive significantly increased the risk of HPV infection. This increase in risk has been reported in the Ethiopia [32] and a number of previous studies [13, 21, 25]. HIV infection causes immune suppression and raises the likelihood of HPV gaining and late clearance [40]. As the clinical staging of people living with HIV advances, the possibility of acquiring HPV infection is higher due to debilitated immunity [41]. The majority of HPV infections are clinically unapparent or asymptomatic in immune-competent individuals; however, immunosuppression caused by HIV seems to favor infection by multiple HPV genotypes, which, if oncogenic, might contribute to the progression of intraepithelial lesions to CIN. The current result suggest that cervical cancer prevention programs in resource-limited settings should highlight inadequate HPV testing resources to HIV-positive clients and those who are likely to have multiple sexual partners, such as sex workers.

## Conclusion

The prevalence of HPV infection among HIV infected women was high in the study area. Given the above associated factors, we recommend the stakeholders to integrate HPV prevention strategies to HIV /AIDS services. Furthermore, the study has provided essential information about the HIV link with hr-HPV infections, which may explain the high prevalence among HIV infected women. This can contribute to policy development and planning of prevention strategies incorporating HPV infection prevention especially among youth and HIV-infected people.

### Electronic supplementary material

Below is the link to the electronic supplementary material.

Supplementary Material 1

## Data Availability

All data are available within the manuscript. Additional data can be obtained from the corresponding author with a reasonable request.

## Abbreviations

AIDS: Acquired Immunodeficiency Syndrome
AOR: Adjusted Odds Ratio
HIV: Human Immunodeficiency Virus
HPV: Human Papilloma Virus
STIs: Sexually Transmitted Infections
WHO: World Health Organization, WLHIV: Women Living with Human Immunodeficiency Virus
