# Supplementary material for: Factors associated with human papillomavirus infections among women living with HIV in public health facilities in Western Oromia, Ethiopia
Source: BMC Womens Health. 2024 Jul 25;24:423. doi: 10.1186/s12905-024-03249-y (PMC11270813; doi:10.1186/s12905-024-03249-y)
Supplement: Supplementary file 1 — Supplementary Material 1 [file 12905_2024_3249_MOESM1_ESM.docx]

**WALLAGAUNIVERSITY**

**INSTITUTE OF HEALTH SCIENCES**

**DEPARTMENT OF PUBLIC HEALTH, EPIDEMIOLOGY TRACK**

**INFORMED CONSENT FORM**

Dear Madam/Ma’am/Missy/Mrs.! ______________________________________

To ensure the health of WLHIV, understanding of the existing health problems and related risky behaviors of these populations are crucial. Owing this, this study is designed to assess the prevalence of HPV and associated factors among WLHIV attending ART service in public health facilities of Western Oromia, Ethiopia.

You are chosen to participate in this study. You being chosen as an individual was done as you are availed to utilize the service the institution is providing. The aim of this study is to assess prevalence of HPV and associated factors to propose future intervention program.

This study questions will involve various personal and sexual issues of individuals. Thus it is your full right to refuse or participate in this study, however, in order to attain the goal of the study, we sincerely request your kindness and good will to participate in the study. Moreover, I assure you that your responses are completely confidential & none of your responses and result of your sample will be reported to anybody else. So, please give me a few minutes to answer the questions and to collect cervical sample for you.

Would you like to participate? **Yes** [___] **No** [___]. Mark**" X"** in the appropriate space.

If **“yes”**, go to the next page. If **“no”**, take your next regular follow up appointment.

Date (DD/MM/YY): _________________

Name of the Health Facility: ___________________

Questionnaire Code: ________________________

Name of data and sample collector: __________________ Signature: _________

Name of supervisor: _____________________ Signature: _______

**English Version Questionnaire**

| **Part I. Questioners for Socio-economic and demographic characteristic data** | | | |
| --- | --- | --- | --- |
| **Code** | **Variables** | **Response** | **Remark** |
| 101 | Age | ___________ |  |
| 102 | Residence | 1. Urban 2. Rural |  |
| 103 | What is your religion? | 1. Orthodox 2. Protestant 3. Muslim 4. Catholic 5. Wakefata 6. Other specify____ |  |
| 104 | What is your ethnic group? | 1. Oromo 2. Amhara 3. Gurage 4. Tigre 5. Other specify______ |  |
| 105 | What is your level of educational? | 1. Never been to school 2. Primary(grade 1-8) 3. Secondary(grade 9-10) 4. High school(grade 11-12) 5. College/University graduate |  |
| 106 | What is your marital status? | 1. Single 2. Married 3. Divorced/separated 4. Widowed |  |
| 107 | Are you employed? | 1. Yes 2. No | If ‘no’ skip to question # 109 |
| 108 | If employed or ‘Yes”, what is your employment? | 1. Government employment 2. Self-employment |  |
| 109 | What is your household annual income? | ________ |  |

| \| **Part II. Questionnaire for Behavior and Age-related characteristics (Sexual History) data:** \| \| --- \| | | | |
| --- | --- | --- | --- | --- |
| 201 | What was your age of menarche? | _____________ |  |
| 202 | What was your age of marriage? | _____________ |  |
| 203 | What was your age of first sexual intercourse? | _____________ |  |
| 204 | What was your age at first pregnancy? | _____________ |  |
| 205 | How many times were you been pregnant? Or number of pregnancies | _____________ |  |
| 206 | Have you ever involved/ experienced missing/Abortion? | 1. Yes 2. No |  |
| 207 | Do you use condom during sexual intercourse? | 1. Yes 2. No | If “No” skip to question # 209 |
| 208 | If ‘yes’, how often do you use condom during sexual intercourse? | 1. Sometimes 2. Always |  |
| 209 | What was the number of life time sexual partners? | 1. 0 B. 1 C. 2 D. 3+ |  |
| 210 | Have you ever had STI in your life (other than HIV)? | 1. Yes 2. No 3. Don’t remember | If no, skip to question # 212. |
| 211 | Have you ever contracted STIs and treated from in the last 12months | 1. Yes 2. No 3. Don’t know |  |
| 212 | Do you have abnormal vaginal discharge?(Self report) | 1. Yes 2. No | If no, skip to question #214 |
| 213 | What is the frequency of the vaginal discharge? (Self-report) | 1. Since last week 2. More than a week 3. Less than 6 month 4. More than or equal to 6month |  |
| 214 | What was your age at first contraceptive use? | ______________ |  |
| 215 | Are you using family planning currently? | 1. Yes 2. No | If no, skip to question # 217. |
| 216 | If yes, what method of contraception do you use? | 1. Implant 2. Tubal ligation 3. IUCD 4. Depo Provera/ Injectable 5. OCP/Pill 6. Condom |  |
| 217 | Do you use chemical products for vaginal care? | 1. Yes 2. No |  |

| **Part III: Questionnaires for substance abuse (Risky Behavior) data:** | | | |
| --- | --- | --- | --- |
| 301 | Do you drink alcohol? | 1. Yes 2. No | If ‘No”, skip to question # 303 |
| 302 | If yes, how often do you drink? | 1. Sometimes 2. Always |  |
| 303 | Do you Smoke Cigarette? | 1. Yes 2. No | If ‘No’, skip to question # 305 |
| 304 | If yes, how often do you Smoke? | 1. Sometimes 2. Always |  |
| 305 | Do you Chew/consume Khat? | 1. Yes 2. No | If ‘no’, skip to question #307 |
| 306 | If yes, how often do you consume khat? | 1. Sometimes 2. Always |  |
| 307 | Do you use Cannabis? | 1. Yes 2. No | If ‘no’, skip to question #309 |
| 308 | If yes, how often do you use Cannabis? | 1. Sometimes 2. Always |  |
| 309 | WHO clinical stage of HIV in life being HIV positive (from Client personal file) | 1. Stage ‘1’ 2. Stage ‘2’ 3. Stage ‘3’ 4. Stage ‘4’ |  |

Principal investigator: Mulatu Abdi, Phone number: +251 917 81 46 54

Data collector name: ______________ Date of Collection: ____________ Signature: ______

**YUUNIVERSIITII WALLAGGAA**

**INISTITIYUUTII SAAYINSIIWWN FAYYAA**

**MUUMMEE FAYYAA HAWAASAA**

**UNKA WALIIGALTEE**

Aadde/Obboleetti: ______________________________________________!

Fayyummaa dubartoota HIV/AIDS qabanii mirkaneessuuf hubannoon fayyummaa isaanii fi amaloota saaxiloo tahanii barbaachisaa dha.

Kaayyoon qo’annoo kanaas dubartoota HIV/AIDS qaban keessatti amaloota saaxiloo tahan fi qabattoota kanaaf murteessoo tahan adda baasuuf dha.

Kanaafuu, qo’annoo kana keessatti akka hirmaattaniif tajaajila argachuuf argamuun keessan akka filatamtan taasiseera.

Kaayyoon qo’annoo kanaa tatamsa’inni dhukuba kaanserii hjummoo gadameessaa ammam baball’atee akka jiruu fi amaloota saalaa dubartoota HIV/AIDS qaban, qabattoota kanaan walitti hidhaman/amaloota fafee, fayyadama kondomii fi gara fuula duraatti akkaataa to’annoo irratti odeeffanno bu’a – qabeessa argamsiisuu dha.

Akkuma beekamu gaaffileen qo’annoo kun dhimmoota dhuunfaa fi saalaa of keessaa qabaa. Kanaafuu, qo’annoo kana keessatti hirmaachuuf ykn dhiisuuf mirga guutuu qabda. Haa tahu malee, galma gahiinsa qo’annoo kanaatiif jecha qo’annoo kana keessatti akka hirmaattaniif fedha keessan isin gaafanna. Qo’annoo kanaaf deebin isin kennitan hunduu icciin isaa guutumaa guutuutti kan eegamu tahuu isiniifan mirkaneessa.

Gaaffilee kennaman isin gaafachuuf yeroo muraasa daqiiqaa 2o hin caalle naaf kennaa!

Hirmaachuuf fedhii qabdu? Eeyyee Lakki

Eeyyee yoo jettan gara gaaffiitti sienna.

Lakki yoo jettan hordoffii itti aanuuf beellama keessan fudhadhaa

Guyyaa (GG/JJ/BB): ______________________________________

Maqaa dhaabbata fayyaa: ______________________________________________________

Koodii Gaaffii: ________________________

Odeeffannoo kan funaanee:_______________Guyyaa funaaname:__________ Mallattoo:_________

Maqaa to’ataa: ___________________ Guyyaa: _____________ Mallattoo: _______

| **Kutaa I: Gaaffiiwwan Seenaa hirmaataa ibsan: Af-gaafii vershinii Afaan Oromoo** | | | |
| --- | --- | --- | --- |
| **Koodii** | **Gaaffii** | **Deebii** | **Yaada** |
| 101 | Umurii | Waggaa_________ |  |
| 102 | Bakka jireenya | 1. Magaalaa 2. Baadiyyaa |  |
| 103 | Amantaan keessan maali? | 1. Ortodooksii 2. Pirootestaantii 3. Musliima 4. Kaatolikii 5. Waaqefataa 6. Kan biro_________ |  |
| 104 | Qomoon keessan maali? | 1. Oromoo 2. Amaara 3. Guraagee 4. Tigiree 5. Kan biro________ |  |
| 105 | Sadarkaan barnoota keessanii maali? | 1. Hin baranne 2. Sadarkaa 1ffaa 3. Sadarkaa 2ffaa 4. Qophaayina 5. Kolleejjii/Yuunivarsiitii |  |
| 106 | Haalli gaa’ela keessanii maali? | 1. Hin heerumne 2. Heerumeera 3. Wal hiikne 4. Abbaan manaa kan irraa du’e |  |
| 107 | Hojii qacaramtanii jirtuu? | 1. Eeyyee 2. Lakki | Lakki yoo ta’e, gaaffii lakk. 109 darbi |
| 108 | Deebiin keessan eeyyee yoo ta’e, haalli qacarrii keessanii maali? | 1. Qacaramaa Mootummaa 2. Qacaramaa Dhuunfaa |  |
| 109 | Galiin maatii keessanii waggaadhaan hangami? | Qarshii____________ |  |

| **Kutaa II. Gaaffilee Amalaa fi Umuriin walqabatan (Sexual History)** | | | |
| --- | --- | --- | --- |
| 201 | Waggaa meeqatti laguu/xurii arguu eegalte? | Waggaa________ |  |
| 202 | Waggaa meeqatti heerumte? | Waggaa_________ |  |
| 203 | Yeroo jalqaba wal quunnamtii saalaa raawwatte umuriin kee meeqa ture? | ______________ |  |
| 204 | Yeroo jalqaba ulfoofte umuriin kee meeqa ture? | Waggaa_________ |  |
| 205 | Si’a meeqa ulfoofte? | _______________ |  |
| 206 | Ulfi sirraa bahe ni beeka? | 1. Eeyyee 2. Lakki |  |
| 207 | Yeroo wal quunnamtii saalaa Kondomii ni fayyadamta? | 1. Eeyyee 2. Lakki | Lakki yoo ta’e, lakk. 209 |
| 208 | Eeyyee yoo ta’e, hagami fayyadamta? | 1. Darbee darbee 2. Yeroo hundumaa |  |
| 209 | Hiriyaa saal quunnamtii meeqa qabda ture? | 1. 0 B. 1 C. 2 D. 3 ol |  |
| 210 | HIV/AIDS alatti jireena keessatti dhibeewwan saal quunnamtiin daddarbaniin qabamtee beekta? | 1. Eeyyee 2. Lakki 3. Hin yaadadhu | Lakki yoo tahe gaaffii lakk 212darbi |
| 211 | Ji’oota 12 n darbanitti yaala dhibeewwan saal quunnamtiin dadddarbanii fudhatteetta? | 1. Eeyyee 2. Lakki 3. Hin beeku |  |
| 212 | Dhangala’aan karaa qaama saalaa /Buqushaa kee bahu ni jira? | 1. Eeyyee 2. Lakki | Yoo lakki ta’e gara gaaffii #214tti darbi |
| 213 | Dhangala’aan kun yoomii kaasee argamuu eegale/hagam ture? | 1. Erga torbee darbeetii 2. Baatii tokkoo ol 3. Baattii jahaa gadi 4. Baatii jahaa fi isaa ol |  |
| 214 | Yeroo mala karoora maatii fayyadamuu eegalte umuriin kee meeqa ture? | ______________ |  |
| 215 | Karoora/qusannaa maatii ni fayyadamta? | 1. Eeyyee 2. Lakki | yoo lakki ta’e gara gaaffii lakk 217 tti darbi |
| 216 | Yoo eeyyee ta’e karoora maati gosa kamiin fayyadamta? | 1. Kan irree keessaa galu 2. Tubal ligation 3. Kangadameessa keessa galu 4. Marfeen kan kennamu 5. Piilsii 6. Kondomii |  |
| 217 | Qulqullina qaama saalaa/Buqushaa kee kunuunsuuf keemikaala adda addaa ni fayyadamta? | 1. Eeyyee 2. Lakki |  |

| **Kutaa III: Gaaffii Amaloota Fafee (Araada) Saaxiloo Tahan (Risky Behavior)** | | | |
| --- | --- | --- | --- |
| 301 | Dhugaatii alkoolii ni dhugdaa? | 1. Eeyyee 2. Lakki | Lakki yoo ta’e,gaafii 303 |
| 302 | Yoo eeyyee ta’e, yero hagamiif dhugda? | 1. Darbee darbee 2. Yeroo hundumaa |  |
| 303 | Tamboo ni xuuxxaa? | 1. Eeyyee 2. Lakki | Lakki yoo ta’e,gaafii 305 |
| 304 | Yoo eeyyee ta’e, yero hagamiif xuuxxa? | 1. Darbee darbee 2. Yeroo hundumaa |  |
| 305 | Jimaa ni qamaata? | 1. Eeyyee 2. Lakki | Lakki yoo ta’e,gaafii 307 |
| 306 | Yoo eeyyee ta’e, yero hagamiif Jimaa qaamta? | 1. Darbee darbee 2. Yeroo hundumaa |  |
| 307 | Qorichoota sammuu nama adoodchan ni fayyadamtaa? | 1. Eeyyee 2. Lakki | Lakki yoo ta’e,gaafii 309 |
| 308 | Yoo eeyyee ta’e, yero hagamiif fayyadamta? | 1. Darbee darbee 2. Yeroo hundumaa |  |
| 309 | Sadarkaa HIV/AIDS (Faayila dhuunfaa maamilaarraa) | 1. Sadarkaa ‘1’ 2. Sadarkaa ‘2’ 3. Sadarkaa ‘3’ 4. Sadarkaa ‘4’ |  |

Qorataa jalqabaa: Mulaatuu Abdii Lakk bilbilaa: +251 917 81 46 54

Odeeffannoo Kan funaanee: _____________Guyyaa: ____________Mallattoo: ________

## Laboratory format: Specimen Transaction, Reception, Storage, Retention and

## Disposal Forms

WALLAGAUNIVERSITY

INSTITUTE OF HEALTH SCIENCES

DEPARTMENT OF PUBLIC HEALTH, EPIDEMIOLOGY TRACK

FORM 1: specimen Transaction Log Sheet

| Serial No. | Specimen ID | Patient ID | Patient name | Sex | Age | Health Facility | Specimen Collection /Receiving | | Test requested | Specimen receiving at laboratory | Signature |
| --- | --- | --- | --- | --- | --- | --- | --- | --- | --- | --- | --- |
|  |  |  |  |  |  |  | Date | Time |  |  |  |
| 1 |  |  |  |  |  |  |  |  |  |  |  |
| 2 |  |  |  |  |  |  |  |  |  |  |  |
| 3 |  |  |  |  |  |  |  |  |  |  |  |
| 4 |  |  |  |  |  |  |  |  |  |  |  |
| 5 |  |  |  |  |  |  |  |  |  |  |  |
| 6 |  |  |  |  |  |  |  |  |  |  |  |
| 7 |  |  |  |  |  |  |  |  |  |  |  |
| 8 |  |  |  |  |  |  |  |  |  |  |  |
| 9 |  |  |  |  |  |  |  |  |  |  |  |
| 10 |  |  |  |  |  |  |  |  |  |  |  |
| Total | |  |  |  |  |  |  |  |  |  |  |

FORM 2: Specimen Receiving Log Sheet

| Serial No. | Patient Name | ID/MRN | Health Facility | Regional Laboratory ID. | Test Ordered | Specimen Type | Specimen Collection | | Transported/collected By | Received by | | Specimen Status | | Initial |
| --- | --- | --- | --- | --- | --- | --- | --- | --- | --- | --- | --- | --- | --- | --- |
|  |  |  |  |  |  |  | Date | Time | Name | Date | Time | Accepted | Rejected |  |
| 1 |  |  |  |  |  |  |  |  |  |  |  |  |  |  |
| 2 |  |  |  |  |  |  |  |  |  |  |  |  |  |  |
| 3 |  |  |  |  |  |  |  |  |  |  |  |  |  |  |
| 4 |  |  |  |  |  |  |  |  |  |  |  |  |  |  |
| 5 |  |  |  |  |  |  |  |  |  |  |  |  |  |  |
| 6 |  |  |  |  |  |  |  |  |  |  |  |  |  |  |
| 7 |  |  |  |  |  |  |  |  |  |  |  |  |  |  |
| 8 |  |  |  |  |  |  |  |  |  |  |  |  |  |  |
| 9 |  |  |  |  |  |  |  |  |  |  |  |  |  |  |
| 10 |  |  |  |  |  |  |  |  |  |  |  |  |  |  |
| Total | |  |  |  |  |  |  |  |  |  |  |  |  |  |

FORM 3: Specimen Storage, Retention and Disposal Form

| Date | Specimen information | | | Time specimen processed | | Specimen processed by | Specimen storage at bench area | | Specimen stored by | If Stored for further use  Refer _____ | If Specimen disposal | | Specimen  disposed by |
| --- | --- | --- | --- | --- | --- | --- | --- | --- | --- | --- | --- | --- | --- |
|  | Test type | total | Lab serial No.(from-to) | from | to |  | Date | Time |  |  | Time | Date |  |
| 1 |  |  |  |  |  |  |  |  |  |  |  |  |  |
| 2 |  |  |  |  |  |  |  |  |  |  |  |  |  |
| 3 |  |  |  |  |  |  |  |  |  |  |  |  |  |
| 4 |  |  |  |  |  |  |  |  |  |  |  |  |  |
| 5 |  |  |  |  |  |  |  |  |  |  |  |  |  |
| 6 |  |  |  |  |  |  |  |  |  |  |  |  |  |
| 7 |  |  |  |  |  |  |  |  |  |  |  |  |  |
| 8 |  |  |  |  |  |  |  |  |  |  |  |  |  |
| 9 |  |  |  |  |  |  |  |  |  |  |  |  |  |
| 10 |  |  |  |  |  |  |  |  |  |  |  |  |  |
| Total | |  |  |  |  |  |  |  |  |  |  |  |  |
